# Optimising Camera Traps for Monitoring Small Mammals

**DOI:** 10.1371/journal.pone.0067940

**Published:** 2013-06-28

**Authors:** Alistair S. Glen, Stuart Cockburn, Margaret Nichols, Jagath Ekanayake, Bruce Warburton

**Affiliations:** 1 Landcare Research, Lincoln, New Zealand; 2 Department of Conservation, Wellington, New Zealand; 3 Biological Sciences, University of Canterbury, Christchurch, New Zealand; Max Planck Institute for Evolutionary Anthropology, Germany

## Abstract

Practical techniques are required to monitor invasive animals, which are often cryptic and occur at low density. Camera traps have potential for this purpose, but may have problems detecting and identifying small species. A further challenge is how to standardise the size of each camera’s field of view so capture rates are comparable between different places and times. We investigated the optimal specifications for a low-cost camera trap for small mammals. The factors tested were 1) trigger speed, 2) passive infrared vs. microwave sensor, 3) white vs. infrared flash, and 4) still photographs vs. video. We also tested a new approach to standardise each camera’s field of view. We compared the success rates of four camera trap designs in detecting and taking recognisable photographs of captive stoats ( *Mustela* *erminea* ), feral cats (*Felis catus*) and hedgehogs ( *Erinaceus* *europaeus* ). Trigger speeds of 0.2–2.1 s captured photographs of all three target species unless the animal was running at high speed. The camera with a microwave sensor was prone to false triggers, and often failed to trigger when an animal moved in front of it. A white flash produced photographs that were more readily identified to species than those obtained under infrared light. However, a white flash may be more likely to frighten target animals, potentially affecting detection probabilities. Video footage achieved similar success rates to still cameras but required more processing time and computer memory. Placing two camera traps side by side achieved a higher success rate than using a single camera. Camera traps show considerable promise for monitoring invasive mammal control operations. Further research should address how best to standardise the size of each camera’s field of view, maximise the probability that an animal encountering a camera trap will be detected, and eliminate visible or audible cues emitted by camera traps.

## Introduction

Currently there are few or no proven techniques for measuring the efficacy of control operations targeting small to medium-sized invasive animals such as stoats ( *Mustela* *erminea* ), feral cats (*Felis catus*) and hedgehogs ( *Erinaceus* *europaeus* ) in New Zealand, especially when these animals are in very low abundance. In many control operations, managers can only assume they have successfully reduced populations of the target species to low levels. For management purposes, indices of relative abundance of pest animals are often sufficient as long as the relationship between the index and density is consistent. Camera traps have demonstrated potential for population assessment, including both relative and absolute density estimates [1–6].

A key challenge in using camera traps to estimate relative abundance of animals is how to standardise the size of each camera’s field of view so that results are comparable between different places and times. This problem has recently been addressed by setting camera traps facing the ground from a fixed height [7], which also results in higher detection probabilities for some species [8]. However, Department of Conservation staff were concerned that cameras deployed in this way would be conspicuous, and therefore vulnerable to theft or vandalism. An alternative is to set cameras close to the ground, align them horizontally, and place a fabric screen at a fixed distance in front of each camera. By ensuring that each camera samples an area of the same size, the number of detections of a species per camera can be used as an index of relative abundance.

Although camera traps have been used mainly to detect relatively large species [4,9,10], recent studies have extended their use to smaller animals such as rodents, small marsupials and lagomorphs [7,11,12]. Detecting small animals raises several challenges. First, small species may be less likely to trigger the passive infrared (PIR) sensors used by most camera traps. Second, identifying small animals from pictures requires them to be photographed at close range, a problem if fast-moving animals cross the camera’s field of view before a photograph is taken. Finally, smaller species may be more difficult to identify from pictures because distinctive features may not be obvious, especially if the animal is partly obscured by vegetation.

We aimed to determine the optimal specifications of a low-cost camera trap for small to medium-sized mammals. The factors tested were: 1) trigger speed, 2) type of sensor (PIR *vs* microwave), 3) flash type (white *vs* infrared), and 4) type of images used (still *vs* video).

## Materials and Methods

### Ethics statement

Care, handling and subsequent euthanasia of animals was carried out in accordance with Landcare Research Standard Operating Procedures, and with approval from the Landcare Research Animal Ethics Committee (Approval No. 13/03/02). All trapping was conducted on the grounds of the Landcare Research animal facility, Lincoln, New Zealand with the permission of the facility manager. The work did not involve endangered or protected species.

### Capture and care of animals

Our trials focused on three priority pest species identified by staff from the New Zealand Department of Conservation: stoats, feral cats and hedgehogs. We used six individuals of each species to sample individual variations in behaviour.

Wild-caught stoats were provided from a captive colony maintained by Landcare Research. Feral cats and hedgehogs were captured in cage traps set on the grounds of the animal research facility. Traps were set in the evening, baited with commercial cat food, and checked shortly after dawn the following morning. Captured animals were acclimatised to captivity for at least 72 hours before being used in trials.

The animals were housed in pens measuring ~5 × 5 × 2 m. They were provided with weatherproof nest boxes and warm bedding, and given drinking water *ad libitum*. Stoats and cats were fed daily on minced meat, cat biscuits or dead day-old chicks. Hedgehogs received similar food, but with eggs in place of day-old chicks.

On completion of the trials, stoats were returned to the captive colony from which they were sourced. Cats and hedgehogs were humanely euthanased by intracardiac injection of pentobarbital following anaesthesia with isofluorane.

### Camera traps

We placed camera traps in each of three video observation pens. Four camera trap designs were used. One was a commercially available trail camera (Reconyx Hyperfire PC900, Reconyx Inc., Holmen). The other three cameras (hereafter referred to as the Jagath 1000A, D, and F) were prototypes built for this study. The specifications of each camera are given in Table 1. We programmed the Reconyx camera to take three photographs each time it was triggered. The Jagath 1000D and F each took a single photograph when triggered, and the Jagath 1000A took a 30-s video clip. Cameras were mounted so that the sensor and lens were approximately 7 cm and 10 cm above the ground, respectively. A shade cloth screen (1 m wide × 40 cm high) was placed 1 m in front of the camera lens to standardise the size of each camera’s field of view. A food lure of minced meat and/or cat biscuits was placed in front of the screen in the middle of the camera’s field of view.

**Table 1:** Specifications of the four camera types used in captive trials. PIR = passive infrared.

	**Camera type**				
	**Reconyx^®^**		**Jagath 1000A**	**Jagath 1000D**	**Jagath 1000F**
**Trigger speed**	0.2 s	2.1 s		1.6 s	1.1 s
**Recovery time**	0.5 s	7 s		7 s	7 s
**Sensor**	PIR	PIR		PIR	Microwave
Horizontal angle of detection*	27°**	42°		35°	−
Vertical angle of detection*	6°**	22°		22°	−
**Light source**	Infrared flash	Infrared LEDs		White flash	White flash
**Flash range**	15 m	10 m		6 m	6 m
**Type of footage**	Still	Video		Still	Still
**Pixel resolution**	2048 x 1536	640 x 480		2560 x 1920	2560 x 1920
**Frames per second**	Up to 2	15		Single shot	Single shot

* Calculated using trigonometry based on detection zones as shown in Figure 2.

** For a camera set at ground height. Due to alignment of sensors the detection zone is wider for a camera mounted higher [20].

To test each camera with each individual animal, the cameras were rotated daily between the three pens. Two cameras (Jagath 1000A and D) were placed side by side in the same pen each night, allowing all four cameras to be tested simultaneously using three pens. Testing was carried out for six nights for each target species, using six individual animals (one night per individual per camera).

### Data collection and analysis

The pens were equipped with Bosch Dinion day/night video cameras (Bosch Security Systems, Sydney), and illuminated at night with infrared light. Continuous video footage was recorded between the hours of 1930 and 0700, beginning 1–2 hrs before dark and finishing 1–2 hrs after sunrise. We used a Geovision-1248 digital video-recording system (Geovision Inc, Taipei) to store and view the date and time stamped footage.

**Figure 1:**
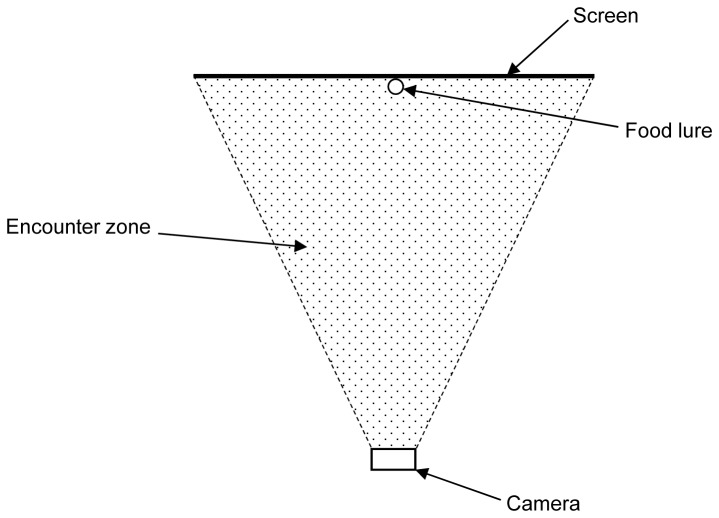
Overhead view of a camera trap showing the triangular zone between the camera and the shade cloth screen. An animal was deemed to have encountered the camera trap if it entered this zone.

When reviewing the continuous video footage, we recorded all encounters between an animal and a camera trap. We defined an encounter as any occasion when any part of an animal entered the triangular area between the camera and the edges of the shade cloth screen (Figure 1). We also noted any obvious reaction by the animal when the camera trap was activated (e.g. suddenly looking at the camera; fleeing). For each encounter we recorded the time of day, the animal’s behaviour (walking, running or paused), if the camera was triggered, and if the animal was clearly identifiable from the resulting photograph or video clip. We then calculated the success rate as the proportion of encounters where the animal was photographed clearly enough to be identified. Continuous video footage showed that animals (usually stoats) running at high speed often triggered the camera traps, but had left the field of view by the time a photograph was taken. These were classed as failed detections, resulting in a low overall success rate for stoats. We therefore calculated a second success rate for encounters in which the animal was not running. Some individuals encountered the camera trap only a few times on a given night. We therefore pooled data for all six individuals of each species to calculate the mean success rate.

We also recorded false triggers, defined as occasions when a camera trap was activated without being encountered by the target animal. Where the likely cause of a false trigger was apparent (e.g. grass moving in strong wind), this was noted. The false trigger rate was calculated for each camera type as the total number of false triggers divided by the total number of occasions on which the camera was activated (including false triggers and real encounters).

Finally, we measured the size of each camera’s detection zone by placing the camera 1 m from a whiteboard marked with 10-cm grid squares. The grid squares extended 50 cm either side of centre, and 40 cm above the height of the sensor (corresponding to the size of the shade cloth screen used in the pen trials). By standing to one side and reaching a hand in front of the whiteboard we were able to trigger the cameras. A hand was reached into each grid square in turn, marking the square with a single pen stroke. By reviewing the resulting images, we determined the area within which the camera detected movement.

## Results

We recorded 1464 encounters with a camera trap by stoats, 393 by cats, and 202 by hedgehogs. Overall success rates for stoats were low, but increased substantially when they were not running (Table 2). Although animals running at high speed were the most common reason for failure to detect stoats during an encounter, long grass may also have contributed to some missed detections. The grass in the observation pens was generally short but, when it grew to ~10 cm tall, stoats were partially concealed by it. The Reconyx PIR sensor took 1–2 s longer to detect stoats in such long grass, and on two occasions the resulting photographs could not be identified because the animal was partly obscured. Both of these were black and white images taken with an infrared flash.

**Table 2:** Success rates in detecting captive stoats (* Mustela erminea *), feral cats (*Felis catus*) and hedgehogs (* Erinaceus europaeus *) encountering four alternative designs of camera trap, and the rate of false triggers for each type of camera.

		**Camera type**			
		**Reconyx^®^**	**Jagath 1000A**	**Jagath 1000D**	**Jagath 1000F**
**Stoats**	Overall success rate	15 (5-90)%	51 (40-90)%	35 (26-90)%	12 (0-25)%
	Success rate when not running	80 (33-100)%	90 (86-100)%	88 (77-100)%	18 (0-31)%
**Cats**	Overall success rate	72 (0-83)%	74 (0-100)%	79 (73-100)%	59 (0-83)%
	Success rate when not running	85 (0-93)%	75 (0-100)%	81 (74-100)%	65 (0-86)%
**Hedgehogs**	Overall success rate	73 (22-100)%	83 (40-100)%	94 (83-100)%	45 (0-90)%
	Success rate when not running	73 (22-100)%	83 (40-100)%	94 (83-100)%	45 (0-90)%
	**False trigger rate**	0.2%	3%	8%	91%

The range of success rates for each camera-species pairing is shown in brackets

Overall success rates were higher for cats than stoats (Table 2). Cats rarely ran across the field of view. Success rates when not running were comparable between cats and stoats, except that the microwave sensor performed better for cats than stoats. Hedgehogs moved more slowly, and were detected at rates comparable to cats (Table 2). However, the cameras were often very slow to detect hedgehogs, which occasionally remained in the centre of the field of view for several minutes before triggering the camera.

The microwave sensor in the Jagath 1000F camera was prone to false triggers, which accounted for over 90% of all photographs taken (Table 2). Many false triggers by the Jagath 1000F were associated with rain or strong wind. The Jagath 1000D had the second highest false trigger rate. However, this was due almost entirely to a large number of false triggers on one windy night. False triggers were rare at other times. The Jagath 1000A and the Reconyx rarely experienced false triggers.

**Figure 2:**
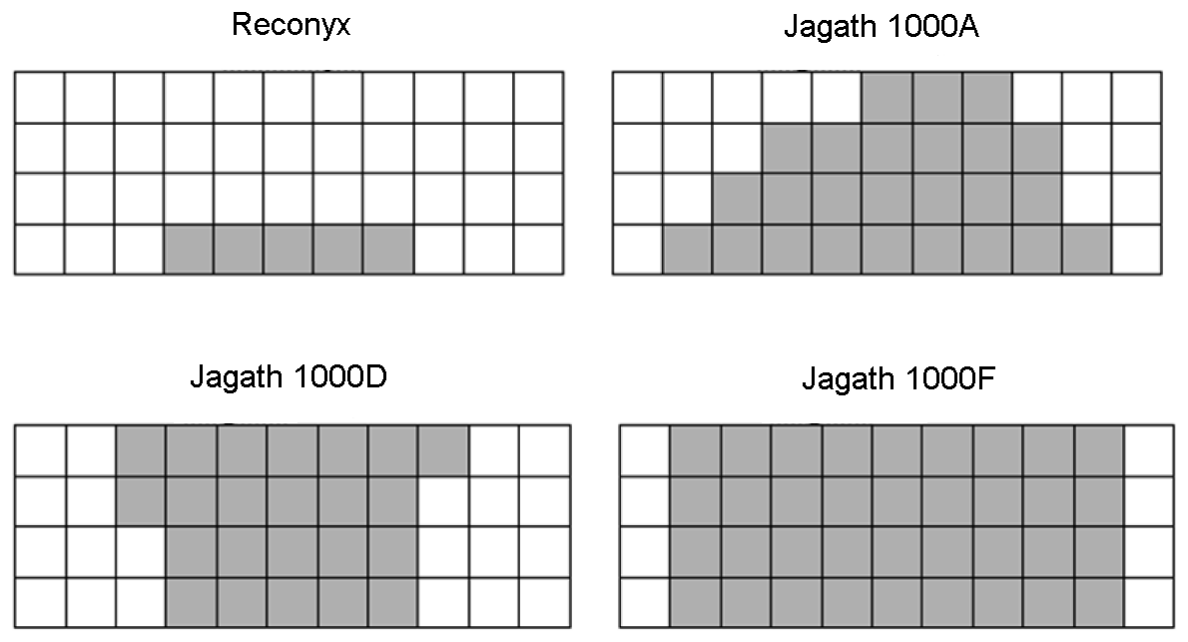
The detection zone of each camera, tested at a distance of 1 m. Shaded squares represent 10 × 10-cm grid squares in which movement triggered the camera.

At a range of 1 m the Reconyx camera had the smallest detection zone, sensing movement 20 cm on either side of centre, and only at a height of 0–10 cm (Figure 2). The Jagath 1000F had the largest detection zone, sensing movement 40 cm on either side of centre, and up to the height of the shade cloth screen. The Jagath 1000A and D had detection zones of intermediate size (Figure 2).

Setting two cameras side by side increased the success rate relative to that of a single camera. For example, pooling the data from the Jagath 1000A and D cameras increased the success rate for cats to 0.93. This is compared to individual success rates of 0.75 and 0.81 for the Jagath 1000A and D, respectively.

### Trigger speed

None of the cameras triggered quickly enough to detect stoats running at high speed. However, three of the four cameras achieved high success rates for stoats that were walking, or that paused in front of the camera (Table 2). These cameras had trigger speeds of 0.2–2.1 s. The only camera that performed poorly for stoats even when not running was the Jagath 1000F, with a trigger speed of 1.1 s. This was due to the poor performance of its microwave sensor (see below).

Trigger speed had little effect on success rates for cats, which rarely passed the camera traps at running speed. The Jagath 1000D (trigger speed 1.6 s) and the Reconyx (trigger speed 0.2 s) achieved similar success rates for cats (Table 2). Trigger speed was unimportant for detecting hedgehogs, which moved more slowly than stoats or cats.

### PIR vs microwave sensor

The microwave sensor in the Jagath 1000F performed poorly in comparison to the PIR sensors used by the other cameras (Table 2). The sensor often failed to trigger when an animal moved in front of it, resulting in low success rates. In addition, it was frequently triggered by rain or wind. The microwave sensor also appeared to lack directionality; the camera was occasionally triggered by animals walking behind it.

### White vs infrared flash

The infrared flash produced black and white (and sometimes blurred) photographs, whereas a white flash produced clear, colour images. Stoats partially outside the camera’s field of view (e.g. when running) were more likely to be identifiable in colour photographs because their black tail tip and sharply contrasting belly fur could be seen, differentiating them from a weasel ( *Mustela* *nivalis* ) or ferret ( *M* *. furo* ) (Appendix S1). For stoats, 33% of photographs taken with an infrared flash could not be clearly identified, compared to 5% for a white flash. Most unclear photographs (92%) were of stoats running at high speed. Cats and hedgehogs were clearly identifiable in photographs regardless of the type of flash used.

Animals frequently reacted to camera traps, although it was not always clear whether to the flash or to a sound emitted by the camera. Stoats frequently reacted to all models of camera, regardless of whether they had a white or infrared flash. Responses included turning the head to look directly at the camera, or moving close to the camera and sniffing it. Stoats did not flee after a camera was triggered or show any subsequent signs of wariness towards the cameras, regardless of flash type.

Three of the six cats showed fright in response to cameras with a white flash. One of the three also showed fright in response to a camera with an infrared flash. Responses included turning quickly and walking back in the direction from which they had come (two cats on four occasions), or rapidly pulling the head back out of the camera’s field of view (one cat on five occasions). On three occasions (once with a white flash and twice with infrared), the cat pulled its head back so quickly that it was not photographed (Appendix S2). This suggests the animal was reacting to a sound made by the camera before the flash was activated. One cat appeared to avoid a camera trap with a white flash after its first encounter. The cat frequently approached the camera’s detection zone but either turned back or changed direction to walk behind the camera. Finally, one cat paused and looked directly at the camera after the infrared flash was triggered, then investigated the camera closely without any signs of wariness.

Hedgehogs showed little obvious reaction to either a white or an infrared flash, although one individual closely investigated a Reconyx camera, repeatedly triggering the infrared flash in the process.

### Video vs still images

Video footage achieved success rates similar to those of still photography (Table 2). On 39 occasions (19%), stoats triggered the camera, left the field of view before the video clip commenced, but then re-entered the frame during the next 30 s while the camera was filming. The same occurred with cats on seven occasions (10%). These animals might not have returned within 30 s if they were not confined to a pen, and would therefore not have been detected. If these events are counted as missed detections, the success rate for stoats using the Jagath 1000A camera becomes 0.47 (overall), and 0.73 (for stoats that were not running). The adjusted success rates for cats are 0.63 (overall) and 0.65 (for cats that were not running).

A 30-s video clip required more memory (10.4 Mb) than still images (~250–750 Kb). Uploading and reviewing video footage was also more time consuming by an order of magnitude.

## Discussion

Camera traps were highly effective at detecting stoats, cats and hedgehogs. Although success rates were relatively low for stoats running at high speed, this behaviour may largely have been an artefact of captivity. Continuous video footage showed that stoats repeatedly ran around the perimeter of their pens at high speed without pausing. This behaviour usually occurred late in the night, after the animal had paused repeatedly in front of the camera to investigate and/or eat the food lure. A stoat in the field would likely be moving at lower speed, and would be more likely to investigate a lure that it had not previously encountered. The success rates calculated for stoats that were not running are probably a more accurate reflection of the success rates that would occur in the field.

A white flash was more effective than infrared for detecting stoats running at high speed. However, this may be unimportant in a field situation, where stoats are unlikely to pass a camera so quickly. The cameras with a white flash appeared more likely to frighten cats, potentially causing subsequent avoidance of camera traps. However, it was not clear whether this effect was due to the flash itself, or to sounds made by the cameras. A further consideration with the white flash is its visibility to the human eye, which may make camera traps more conspicuous in the field. Cameras with an infrared flash may be less likely to be stolen or vandalised.

Video footage did not have any advantages over still photography in detecting and allowing identification of the target species. Given the increased memory requirements and processing time for video, it is clearly less efficient than still images. Video cameras may also be less suitable for long deployments as the large files may rapidly fill the camera’s memory card. However, a video camera similar to the Jagath 1000A might be useful when the objective is not simply to detect animals but also to observe behaviour, for example, in studies of nest predation [13] or bait removal [14].

Our results suggest that cameras do not need a trigger speed as fast as some commercially available models, provided the target animals do not run past the camera at high speed. A trigger speed of 1.6 s was sufficient to photograph 80-90% of animals that walked past the Jagath 1000D camera or paused in front of it. The detection zones of all cameras were narrower than the width of the shade cloth screen. Placing cameras slightly further from the screen and/or modifying the arrangement of the PIR sensor(s) may result in a wider detection zone, and hence earlier detection of animals as they move into the camera’s field of view. This may allow animals running at higher speeds to be photographed clearly. Nelson and Scroggie [11] recommend a distance of ~1.5 m from the camera to the target area for detecting small animals.

Hedgehogs never crossed the camera’s field of view at high speed. However, the cameras were often very slow to detect hedgehogs, sometimes taking several minutes before triggering. The hedgehog’s spines and under-fur presumably mask its body heat from the PIR sensor. Hedgehogs that paused to feed in front of the camera without being detected often triggered the camera when they began to move away again. Thus, although cameras were slow to respond, few detections were missed.

The microwave sensor was clearly less effective than PIR sensors. It was less successful at detecting animals, and also had a false trigger rate > 90%. With the exception of one night when the Jagath 1000D was triggered over 40 times by strong wind, the other cameras tested in this trial had false trigger rates < 10%. In comparison, De Bondi et al. [7] reported a false trigger rate of 11%.

As the next step, field trials are needed to compare the results of camera traps with those of existing methods such as tracking tunnels [15] for detecting stoats, cats and hedgehogs. A camera trap to detect these species should use a PIR sensor, take still images, and have a trigger speed of no more than 1.6 s. Field trials should initially use cameras with an infrared flash. However, if some images cannot be identified (e.g. because the animal is partly outside the field of view or obscured by vegetation), cameras with a white flash should be considered. Camera traps should be deployed so as to standardise the size of the detection zone. This can be achieved by vertical orientation of the camera [7,8], or by using a fabric screen, and further testing is needed to determine optimal dimensions (e.g. placing the screen slightly further from the camera would widen the detection zone, but may also increase the chances of false triggers).

An alternative approach would be to replace the fabric screen with markers (either on the ground, incorporated into the camera’s viewfinder, or using software) to delineate the target zone. Images of animals outside the marked zone could be disregarded for estimating abundance, but might still provide useful information on presence/absence. However, this would require that the cameras emit no light or sound detectable by the target animals. In the absence of a fabric screen, animals approaching from directly in front may trigger the camera before they enter the designated target zone. Any visible flash or audible sound at this time could frighten wary individuals and/or attract inquisitive ones. Such behaviours potentially exacerbate variation in capture probability between individual animals, and between subsequent encounters with the same individual, both of which may bias monitoring results. Light produced by infrared flashes can extend into the spectrum visible to humans and/or other mammals [16–19]. One solution may be to place a filter over the infrared flash, ensuring that no visible light passes through.

Camera traps for monitoring stoats, cats and hedgehogs should use a lure in the centre of the camera’s field of view. This should not only increase the encounter rate of animals with the traps, but also encourage animals to pause in front of the camera, increasing the success rate. The detection zone should have low vegetation cover (< 10 cm) so that small animals are detected by the camera’s sensor and photographed clearly.

Camera traps show considerable promise as a simple and inexpensive method to monitor the effectiveness of invasive mammal control operations. Further research and development should address how best to standardise the size of each camera’s field of view, maximise the probability that an animal encountering a camera trap will be detected, and eliminate visible or audible cues emitted by camera traps.

## Supporting Information

Appendix S1 Photographs of stoats ( *Mustela* *erminea* ) running at high speed past a camera trap. When an animal was partly outside the field of view, colour photographs taken with a white flash were more readily identified to species than black and white photographs obtained with an infrared flash.
(DOCX)

Appendix S2 Video footage of a cat reacting to, and subsequently avoiding, a camera trap. The camera trap captured a photograph of the animal on the first encounter shown, but not the second.
(MPG)

## References

[1] Rowcliffe JM, Field J, Turvey ST, Carbone C (2008) Estimating animal density using camera traps without the need for individual recognition. J Appl Ecol 45: 1228-1236. doi:10.1111/j.1365-2664.2008.01473.x.

[2] Royle JA, Karanth KU, Gopalaswamy AM, Kumar NS (2009) Bayesian inference in camera trapping studies for a class of spatial capture-recapture models. Ecology 90: 3233-3244. doi:10.1890/08-1481.1. PubMed: 19967878. 19967878 10.1890/08-1481.1

[3] Bengsen AJ, Leung LK-P, Lapidge SJ, Gordon IJ (2011) Using a general index approach to analyze camera-trap abundance indices. J Wildl Manag 75: 1222-1227. doi:10.1002/jwmg.132.

[4] O’Brien TG, Kinnaird MF, Wibisono HT (2003) Crouching tigers, hidden prey: Sumatran tiger and prey populations in a tropical forest landscape. Anim Conserv 6: 131-139. doi:10.1017/S1367943003003172.

[5] Noss AJ, Gardner B, Maffei L, Cuéllar E, Montaño R et al. (2012) Comparison of density estimation methods for mammal populations with camera traps in the Kaa-Iya del Gran Chaco landscape. Anim Conserv 15: 527-535. doi:10.1111/j.1469-1795.2012.00545.x.

[6] Lyra-Jorge MC, Ciocheti G, Pivello VR, Meirelles ST (2008) Comparing methods for sampling large- and medium-sized mammals: camera traps and track plots. Eur J Wildl Res 54: 739-744. doi:10.1007/s10344-008-0205-8.

[7] De Bondi N, White JG, Stevens M, Cooke R (2010) A comparison of the effectiveness of camera trapping and live trapping for sampling terrestrial small-mammal communities. Wildl Res 37: 456-465. doi:10.1071/WR10046.

[8] Smith JK, Coulson G (2012) A comparison of vertical and horizontal camera trap orientations for detection of potoroos and bandicoots. Aust Mammal 34: 196-201. doi:10.1071/AM11034.

[9] Karanth KU (1995) Estimating tiger *Panthera tigris* populations from camera-trap data using capture-recapture models. Biol Conserv 71: 333-338. doi:10.1016/0006-3207(94)00057-W.

[10] Karanth KU, Nichols JD (1998) Estimation of tiger densities in India using photographic captures and recaptures. Ecology 79: 2852-2862. doi:10.1890/0012-9658(1998)079[2852:EOTDII]2.0.CO;2.

[11] Nelson JL, Scroggie MP (2009) Remote cameras as a mammal survey tool: survey design and practical considerations. Arthur Rylah Institute for Environmental Research unpublished report number 2009/36. Heidelberg: Department of Sustainability and Environment.

[12] Latham ADM, Nugent G, Warburton B (2012) Evaluation of camera traps for monitoring European rabbits before and after control operations in Otago, New Zealand. Wildl Res 39: 621-628.

[13] Sanders MD, Maloney RF (2002) Causes of mortality at nests of ground-nesting birds in the Upper Waitaki Basin, South Island, New Zealand: a 5-year video study. Biol Conserv 106: 225-236. doi:10.1016/S0006-3207(01)00248-8.

[14] Glen AS, Dickman CR (2003) Monitoring bait removal in vertebrate pest control: a comparison using track identification and remote photography. Wildl Res 30: 29-33. doi:10.1071/WR01059.

[15] Blackwell GL, Potter MA, McLennan JA (2002) Rodent density indices from tracking tunnels, snap-traps and Fenn traps: do they tell the same story? N Z J Ecol 26: 43-51.

[16] Innes JG, Crook B, Jansen P (1994) A time-lapse video camera system for detecting predators at nests of forest birds: a trial with North Island kokako. Proceedings of the Resource Technology Conference: Melbourne University of Melbourne pp. 439–448.

[17] Brown KP, Moller H, Innes J, Jansen P (1998) Identifying predators at nests of small birds in a New Zealand forest. Ibis 140: 274-279.

[18] Meek P, Fleming P, Ballard G (2012) An Introduction to Camera Trapping for Wildlife Surveys in Australia. Canberra: Invasive Animals CRC.

[19] Newbold HG, King CM (2009) Can a predator see 'invisible' light? Infrared vision in ferrets (*Mustela furo*). Wildl Res 36: 309-318. doi:10.1071/WR08083.

[20] Reconyx (2011) Hyperfire High Performance Cameras Instruction Manual. Holmen: Reconyx

