# Supplementary material for: Optimising Camera Traps for Monitoring Small Mammals
Source: PLoS One. 2013 Jun 28;8(6):e67940. doi: 10.1371/journal.pone.0067940 (PMC3695914; doi:10.1371/journal.pone.0067940)
Supplement: Appendix S1 — When an animal was partly outside the field of view, colour photographs taken with a white flash were more readily identified to species than black and white photographs obtained with an infrared flash. (DOCX) [file pone.0067940.s001.docx]

**Supporting information for**

# Optimising camera traps for monitoring small mammals

A. S. Glen, S. Cockburn, M. Nichols, J. Ekanayake & B. Warburton

**Appendix S1**

(a) (b)


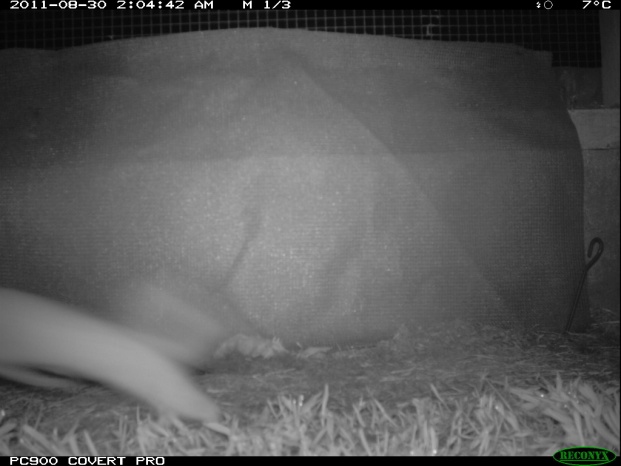

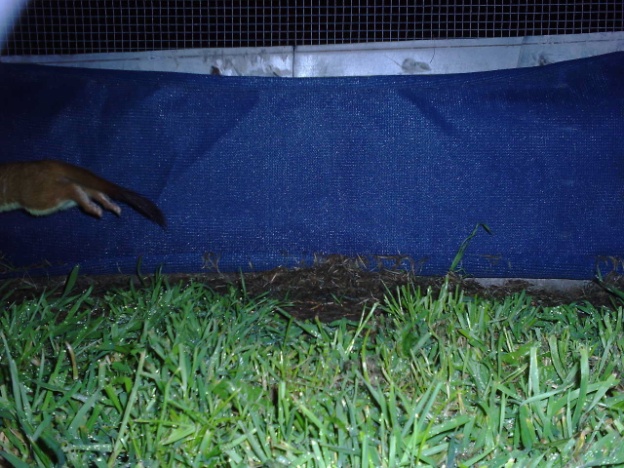


Images of stoats (*Mustela erminea*) taken with an infrared flash (a) and a white flash (b). Both animals are partly outside the camera’s field of view but the clear, colour image obtained with a white flash allows easy identification to species.
